# Coumarin and Its Derivatives—Editorial

**DOI:** 10.3390/molecules26206320

**Published:** 2021-10-19

**Authors:** Maria João Matos

**Affiliations:** 1 CIQUP/Department of Chemistry and Biochemistry, Faculty of Sciences, University of Porto, 4169-007 Porto, Portugal; maria.matos@fc.up.pt or mariajoao.correiapinto@usc.es; 2 Departamento de Química Orgánica, Facultade de Farmacia, Universidade de Santiago de Compostela, 15782 Santiago de Compostela, Spain

Coumarins are widely distributed in nature and can be found in a large number of naturally occurring and synthetic bioactive molecules [1]. The unique and versatile oxygen-containing heterocyclic structure makes them a privileged scaffold in Medicinal Chemistry [1]. The large-conjugated system, with electron-rich and charge-transport properties, is important for the interaction of this scaffold with other molecules and ions [1]. Therefore, many coumarin derivatives have been extracted from natural sources, designed, synthetized, and evaluated on different pharmacological targets [2]. In addition, coumarin-based ion receptors, fluorescent probes, and biological stains are growing quickly and have extensive applications to monitor timely enzyme activity, complex biological events, as well as accurate pharmacological and pharmacokinetic properties in living cells [3]. The extraction, synthesis, and biological evaluation of coumarins have become extremely attractive and rapidly developing topics. A large number of research and review papers compile information on this important family of compounds in 2020 [3]. Research articles, reviews, communications, and concept papers focused on the multidisciplinary profile of coumarins, highlighting natural sources, most recent synthetic pathways, along with the main biological applications and theoretical studies, were the main focus of this Special Issue.

The anticoagulatory activity of coumarins is one of the most classic applications of this family of compounds, acenocoumarol and warfarin being the most important approved drugs. The use of one or another depends on different factors. However, the real evidence on their different results is not completely clear. Therefore, the clinical results for both molecules were studied on 2111 MPHV patients included in the nationwide PLECTRUM registry [4]. In addition, the antiplatelet aggregation profile of coumarin, esculetin and esculin, were determined by studying cyclooxygenase I (COX-I) inhibition [5].

Inflammation is another area of constant interest. Hydroxycoumarins are on the top of the list, 4-hydroxy-7-methoxycoumarin being described as an inhibitor of inflammation in LPS-activated RAW264.7 macrophages by suppressing the nuclear factor kappa B (NF-κB) and MAPK activation [6]. This simple coumarin reduced the production of nitric oxide (NO), prostaglandin E2 (PGE2), proinflammatory cytokines such as tumor necrosis factor (TNF)-α, interleukin (IL)-1β and IL-6, and the expression of inducible nitric oxide synthase (iNOS) and cyclooxygenase 2 (COX-2), being non-cytotoxic for different cell lines. Moreover, this molecule decreased phosphorylation of extracellular signal-regulated kinase (ERK1/2) and c-Jun N-terminal kinase/stress-activated protein kinase (JNK), but not that of p38 MAPK [6]. In addition, coumarins have been described as anti-inflammatory and antioxidant compounds with a potential action in inflammatory bowel disease [7]. These molecules display a protective action in intestinal inflammation by modulating different mechanisms and signaling pathways, mainly modulating immune and inflammatory responses, and protecting against oxidative stress.

Neurodegenerative diseases are another classical application of coumarins in drug discovery. The design of new hybrids, especially looking for a multitarget function, is a trend strategy. Coumarin-chalcone hybrids have been described as potent and selective monoamine oxidase B (MAO-B) inhibitors [8]. A series of fourteen new derivatives were described, an IC_50_ in the nanomolar range presenting the best compound. Theoretical approaches corroborated the interaction and selectivity of these compounds for the B isoform. Coumarin-chalcone hybrids also attracted the attention by being adenosine receptor modulators [9]. This family of G-protein-coupled receptors (GPCRs) is especially important in neurological and psychiatric disorders such as Parkinson’s and Alzheimer’s diseases, epilepsy, and schizophrenia. The studied series proved to be interesting for the design of potent and selective human A_1_ or A_3_ ligands. In general, molecules bearing hydroxy groups showed more A_1_ affinity, while the methoxy counterparts showed A_3_ selectivity. On the other hand, extracts from plants and their isolated compounds are also being used as inhibitors of enzymes involved on neurodegenerative diseases. Coumarin glycyrol and liquiritigenin, isolated from *Glycyrrhiza uralensis*, were the most promising molecules [10]. The first one proved to inhibit butyrylcholinesterase (BuChE), acetylcholinesterase (AChE) and MAO-B in the micromolar range, being reversible and noncompetitive inhibitors of BuChE. The second one proved to be reversible and competitive with MAO-B inhibitor in the nanomolar range. Finally, curcumin–coumarin hybrids have been also described as multitarget agents against neurodegenerative disorders [11]. From the studied series, most of the 3-(7-phenyl-3,5-dioxohepta-1,6-dien-1-yl)coumarins proved to be moderate inhibitors of *h*MAO, AChE, and BuChE, also displaying antioxidant activity (scavenging DPPH free radical). Two compounds out of this series also showed neuroprotective activity against hydrogen peroxide (H_2_O_2_) in the SH-SY5Y cell line. The formulation of these derivatives in nanoparticles improved this last property.

Anticancer activities for coumarins have been also reported. Coumarin-3-carboxamide derivatives have been reported, and 4-fluoro and 2,5-difluoro benzamides presented activities against HepG2 and HeLa cancer cell lines comparable to doxorubicin, exhibiting low cytotoxicity against LLC-MK2 normal cell line [12]. From the combination of simple coumarins (osthole, umbelliferone, esculin or 4-hydroxycoumarin) with sorafenib, an antiglioma compound was also reported by studying human glioblastoma multiforme (T98G) and anaplastic astrocytoma (MOGGCCM) cells lines [13]. 

Psoralen derivatives with electrophilic warhead variations at position 3 have been described for their immunoproteasome inhibitory activity [14]. The studied compounds proved to be slightly less active inhibiting the β5i subunit of immunoproteasome than the previously reported 7*H*-furo[3,2-g]chromen-7-one (psoralen)-based compounds with an oxathiazolone warhead. These results allowed to establish important structure–activity relationships that will guide the design of potent and selective immunoproteasome inhibitors. 

As said before, several coumarins are naturally occurring molecules. Therefore, there is intensive research on plants and extracts analysis. Sixty coumarin derivatives from *Artemisia capillaris* were studied for their constitutive androstane receptor (CAR) activation [15]. Amongst all the molecules studied in the in vitro CAR activation screening, 6,7-diprenoxycoumarin proved to be the most interesting for further studies. A review paper on the natural occurrence, biosynthesis, and biological properties of two 3-prenylated coumarins has been described [16]. A dihydrofuranocoumarin (chalepin) and furanocoumarin (chalepensin) are in the focus of this overview. They were isolated from the first time from the medicinal plant *Ruta chalepensis* L. (Fam: Rutaceae) but are also present in species of the genera Boenminghausenia, Clausena, and Ruta. These two natural products have been described for their anticancer, antidiabetic, antifertility, antimicrobial, antiplatelet aggregation, antiprotozoal, antiviral, and calcium antagonistic properties. The same group focused a second review on the natural origin, biosynthesis, and pharmacological activities of tetracyclic 4-substituted dipyranocoumarins, the calanolides [17]. Ultra-high-performance liquid chromatography coupled with a mass spectrometry (UHPLC-MS) methodology has been used for identifying and quantifying coumarins from a group of twenty-eight plants (roots and leaves) from Arabidopsis natural populations [18]. Simple coumarins such as scopoletin, umbelliferone and esculetin, along with their glycosides scopolin, skimmin and esculin, respectively, have been identified. Finally, the ability of different coumarins to inhibit quorum sensing when combined with small plant-derived molecules identified in various plants extracts has been described [19].

The development of new chemical tools and strategies to obtain different coumarins, and the update of the traditional ones, are a continuous field of research. Chiral tertiary amine catalyzed asymmetric [4 + 2] cyclization of 3-aroylcoumarins with 2,3-butadienoate has been described [20]. Two reviews on the synthetic strategies to obtain coumarin(benzopyrone)-fused five-membered aromatic heterocycles built on the α-pyrone moiety, one centered on five-membered aromatic rings with a single heteroatom and the other one with multiple heteroatoms, have also been published [21,22]. New 3-ethynylaryl coumarin-based dyes for DSSC applications were included in this monographic issue [23]. The synthetic pathways, spectroscopic properties and theoretical calculations were included. The structural characterization (UV-Visible spectroscopy, thermal analysis by differential scanning calorimetry and TGA, ^1^H NMR and X-ray diffraction) of mono and dihydroxylated umbelliferone derivatives has been also described [24]. 3-Carboxylic acid and formyl-derived coumarins have been proposed as photoinitiators in the photo-oxidation or photo-reduction processes for photopolymerization upon visible light [25]. These characteristics are related to the potential of these molecules in the photocomposite synthesis and 3D printing applications [25]. Finally, *in silico* tools (i.e. MetFrag, SIRIUS version 4.8.2, CSI:FingerID and CANOPUS) have been used for the structural elucidation of ferulenol, synthetized by engineered *Escherichia coli* [26]. This study highlights the importance of 4-hydroxycoumarins as lead molecules for the chemical synthesis of several bioactive compounds and drugs.

The huge and growing range of applications of coumarins described in this Special Issue is a demonstration of the potential of this family of compounds in Organic Chemistry, Medicinal Chemistry, and different sciences related to the study of natural products. This Special Issue includes 24 articles: 18 original papers and 6 review papers. The versatility of this scaffold is also being demonstrated by the number of manuscripts revealing and highlighting its potential. Based on the current results, it may be expected that the utility of coumarins as scaffolds for drug design, as structures for chemical synthesis and as fluorescent probes, may grow in the next years. Finally, it seems that simple coumarins are still more explored than complex derivatives. 
